# Natural products for the management of diarrhea: a systematic review of clinical efficacy, safety, and certainty of evidence

**DOI:** 10.3389/fphar.2026.1810632

**Published:** 2026-08-26

**Authors:** Camila Bach, Carolina Silva Schiebel, Lara Luisa Valerio de Mello Braga, Ana Carolina Vieira Ulysséa Fernandes, Mariana Avancini, Gabriel Ferreira Fernandes, Jonatan Zaleski, Jessica Boschini D’Agostin, Daniele Maria-Ferreira

**Affiliations:** 1 Faculdades Pequeno Príncipe, Curitiba, Paraná, Brazil; 2 Instituto de Pesquisa Pelé Pequeno Príncipe, Curitiba, Paraná, Brazil

**Keywords:** diarrhea, diarrhea management, evidence certainty, herb-drug interactions, oncology supportive care

## Abstract

**Introduction:**

Diarrhea is a common gastrointestinal disorder caused by infections, inflammatory diseases, or treatment‐related toxicity, particularly from chemotherapy and radiotherapy, often compromising treatment adherence and quality of life. Natural products have been increasingly investigated as complementary therapeutic strategies; however, their clinical efficacy, safety, and certainty of evidence remain unclear.

**Methods:**

This systematic review evaluated clinical efficacy, safety, and certainty of evidence of natural products for the management of diarrhea in oncological and non‐oncological settings. The review followed PRISMA guidelines and was registered in PROSPERO (CRD42025572477). PubMed, MEDLINE, and the Cochrane Library were systematically searched to identify human clinical trials evaluating natural products for diarrhea. Certainty of evidence was assessed using the GRADE approach.

**Results and Discussion:**

Ten studies met the inclusion criteria and evaluated interventions including Aloe vera (L.) Burm. f [Asphodelaceae; Aloe barbadensis], partially hydrolyzed guar gum, Berberis aristata DC [Berberidaceae], and multibotanical herbal formulations such as Entoban and Amoebex. Most studies reported significant improvements in diarrhea-related outcomes, including reduced stool frequency and severity, improved stool consistency, and relief of associated gastrointestinal symptoms. An exploratory narrative synthesis suggested context-specific mechanisms of action, with microbiota modulation and anti-inflammatory effects more frequently associated with treatment-related diarrhea, whereas antimicrobial activity and intestinal barrier support appeared more relevant in nononcological conditions. However, the certainty of evidence was predominantly low to very low because of methodological limitations, including small sample sizes, inadequate blinding, heterogeneous outcome measures, and reliance on single-study evidence. Safety reporting was generally insufficient, although most interventions were associated with few reported adverse events. Complementary pharmacological evidence was used to contextualize potential herb-drug interactions, highlighting important safety considerations, particularly for patients receiving anticancer therapies with narrow therapeutic indices. Overall, natural products show promise as supportive interventions for diarrhea management; however, the current evidence is insufficient to support definitive clinical recommendations. High-quality randomized controlled trials with standardized outcome measures and comprehensive safety assessments are needed to establish their therapeutic value.

**Systematic Review Registration:**

https://www.crd.york.ac.uk/PROSPERO/view/CRD42025572477.

## Introduction

Diarrhea is a common gastrointestinal disorder characterized by increased frequency of bowel movements and decreased stool consistency, resulting in loose or watery stools (Schiller et al., 2017; Meisenheimer et al., 2022). It can be a symptom of a variety of underlying conditions and can last for several days depending on the cause (Arasaradnam et al., 2018; Black et al., 2020). The etiology of diarrhea is diverse and may include infectious agents (viral, bacterial or parasitic), adverse drug reactions, inflammatory bowel disease, food intolerances, endocrine disorders and malabsorption syndromes (Parvaneh et al., 2019; Ferris et al., 2023). Given its multifactorial nature and potential to cause dehydration, electrolyte imbalances and nutritional deficiencies, diarrhea remains an important clinical problem in both acute and chronic settings (Shane et al., 2017).

**FIGURE 1:**
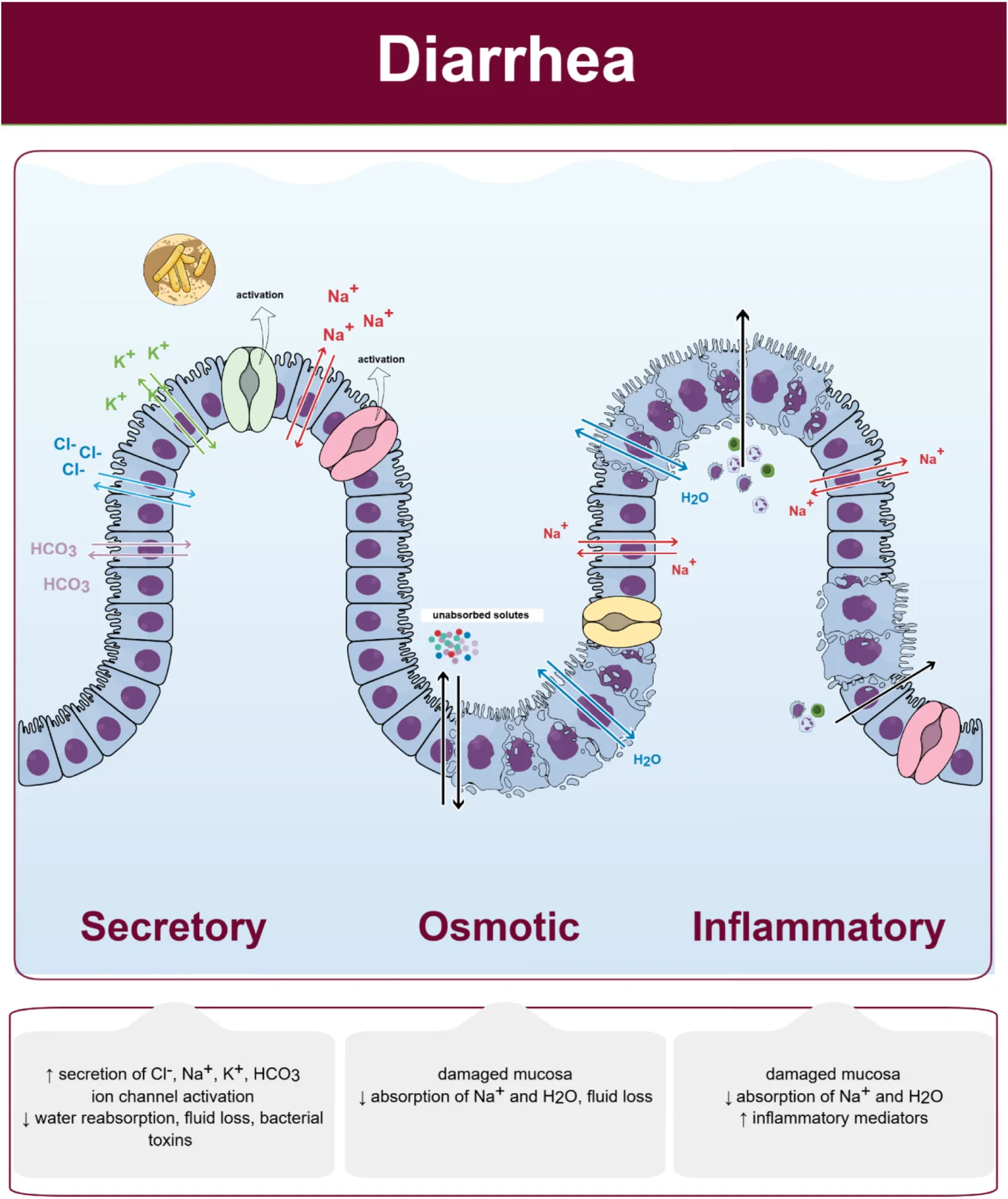
Mechanisms of diarrhea. Schematic overview of the main pathophysiological mechanisms of diarrhea, including secretory diarrhea (increased active secretion of water and electrolytes), osmotic diarrhea (presence of non-absorbed solutes creating an osmotic gradient), and inflammatory diarrhea (mucosal damage with increased permeability and fluid exudation).

Diarrhea can be classified into three main mechanisms (Figure 1). In secretory diarrhea, there is increased secretion of water and electrolytes into the intestinal lumen independent of food intake, usually associated with bacterial toxins (Moon et al., 2015). Osmotic diarrhea results from unabsorbed solutes that draw water into the intestine, often due to disruption of the mucosal barrier, leading to impaired absorption and fluid imbalance (Zhang et al., 2024). Inflammatory diarrhea or exudative diarrhea is due to mucosal damage and the release of inflammatory mediators; it is typically accompanied by fever and abdominal pain and often occurs in invasive infections and inflammatory bowel disease, and stools may contain red or white blood cells (Camilleri, 2015; Sokic-Milutinovic et al., 2022). Although these represent the main pathophysiological mechanisms, diarrhea may also result from impaired nutrient absorption and alterations in intestinal motility, which can contribute to or overlap with the mechanisms described above (Juckett and Trivedi, 2011).

In the clinical context, understanding the underlying mechanisms of different types of diarrhea is essential for identifying the pathways through which natural products may exert protective and modulatory effects (Camilleri, 2015; Moon et al., 2015; Sokic-Milutinovic et al., 2022; Zhang et al., 2024). Natural products with antidiarrheal activity modulate the enteric nervous system, reducing intestinal motility (Yu et al., 2019). Additionally, multiple bioactive compounds have demonstrated the ability to inhibit inflammatory mediators, directly counteracting the mucosal damage observed in inflammatory diarrhea (Ried et al., 2020; Giacosa et al., 2022). In antineoplastic treatment-induced diarrhea, natural products may also help preserve intestinal barrier integrity and modulate the gut microbiota (Kono et al., 2023), potentially mitigating the adverse effects of conventional therapies and improving the quality of life for patients undergoing chemotherapy (Sahebnasagh et al., 2017; Ried et al., 2020).

Assessment of acute diarrhea should focus on signs of dehydration, blood in the stool, fever, and vomiting to guide diagnosis and fluid management (Barr and Smith, 2014). Most cases resolve without testing, although stool parasitology is indicated for persistent symptoms and cultures for severe or dysenteric cases (Shane et al., 2017). In chronic diarrhea, history is critical, including stool characteristics, pain, weight loss, diet, and medications (Arasaradnam et al., 2018). Basic laboratory tests evaluate anemia and inflammation, with stool tests for fecal fat and parasites. Further investigations may include calprotectin, thyroid function, celiac serology, and colonoscopy if required (Sokic-Milutinovic et al., 2022).

Treatment of acute diarrhea primarily focuses on fluid and electrolyte replacement, usually with oral rehydration solutions or, in severe cases, intravenous fluids (Corinaldesi et al., 2012; Brandt et al., 2015). Treatment of chronic diarrhea depends on the underlying cause. Symptomatic therapies, including loperamide and diphenoxylate, are commonly used to reduce stool frequency but do not address underlying inflammatory conditions such as inflammatory bowel disease. In functional disorders such as irritable bowel syndrome with diarrhea (IBS-D), microbiota-targeted therapies, including rifaximin, may be considered. Randomized controlled trials and meta-analyses have demonstrated modest but clinically meaningful efficacy for these therapies (Pimentel et al., 2011; Menees et al., 2012; Riddle et al., 2016).

Natural products have emerged as potential alternatives for the treatment of diarrhea, as they are often associated with a lower incidence of adverse effects, such as dry mouth and headaches, which are commonly observed with conventional antidiarrheal agents, potentially improving patient outcomes (Ried et al., 2020). In this context, this systematic review aims to evaluate and summarize clinical evidence on the use of natural products for the treatment of diarrhea associated with antineoplastic therapy and diarrhea occurring in its absence.

## Methodology

This systematic review was conducted in accordance with the PRISMA (Preferred Reporting Items for Systematic Reviews and Meta-Analyses) guidelines to ensure methodological rigor and transparency. The review protocol was prospectively registered in the International Prospective Register of Systematic Reviews (PROSPERO) under registration number CRD42025572477 to improve reproducibility and minimize reporting bias. The PICO (population, intervention, comparison, and outcome) strategy was used to guide the development of the research question. The population (P) included individuals undergoing antineoplastic treatment and suffering from diarrhea, as well as individuals without antineoplastic therapy who had gastrointestinal disorders of different etiologies. The intervention (I) was the use of natural products, while the comparison (C) included healthy individuals, untreated groups, or positive control groups. The outcome (O) was the potential reduction of diarrheal diseases. Based on these elements, the guiding question was formulated as follows: Do natural products improve diarrhea in individuals with and without antineoplastic treatment?

### Search strategy and study selection

The initial literature search was conducted on 27 November 2023, in the PubMed, Medline, and Cochrane databases. To optimize the results, different combinations of descriptors were used in each database. The descriptors for the PubMed search were (diarrhea [title/abstract]) OR (dysentery [title/abstract]) AND (natural products [title/abstract]) OR (natural compounds [title/abstract]) OR (herbal products [title/abstract]) AND (chemotherapy [title/abstract]) OR (antineoplastics [title/abstract]) AND (cancer [title/abstract]). In Medline, the descriptors were (diarrhea) OR (dysentery) AND (natural products) OR (natural compounds) AND (chemotherapy) OR (antineoplastics). In the Cochrane database, the descriptors were (diarrhea):ti,ab,kw AND (natural products):ti,ab,kw OR (natural compounds):ti,ab,kw AND (chemotherapy):ti,ab,kw AND (cancer):ti,ab,kw. Articles published between 2013 and 2023 (10 years) were included. The search was updated on 1 April 2025, by manually screening for new publications using the same descriptors. The Rayyan artificial intelligence platform was used to support article search and selection. At least three authors performed these steps, and disagreements were resolved by the corresponding author (D.M.-F.).

### Inclusion and exclusion criteria

**FIGURE 2:**
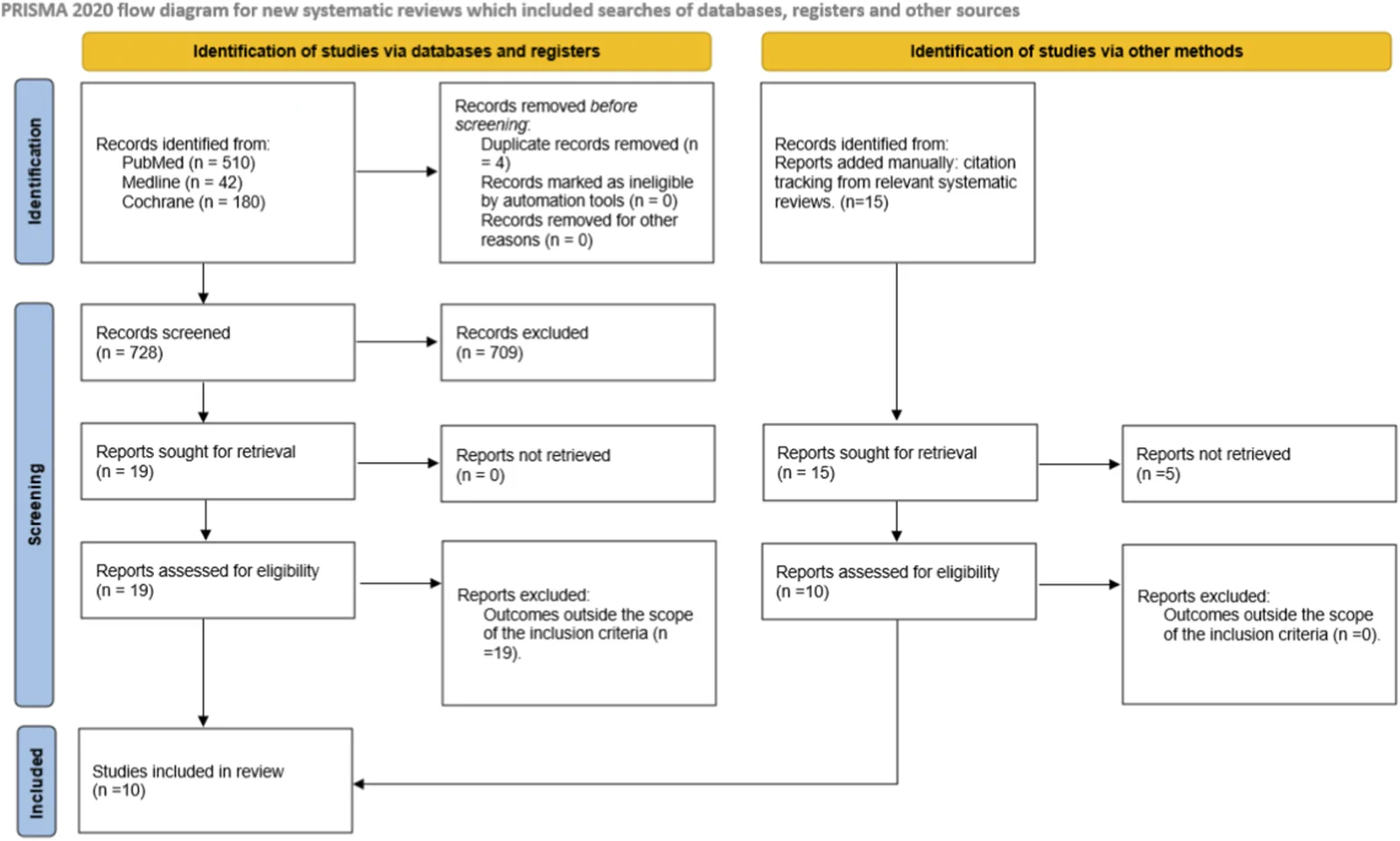
Prisma (preferred reporting items for systematic reviews and meta-analyses) flowchart of the organized literature review.

The articles selected for this review focused on the use of natural products and their beneficial effects in the treatment of diarrhea. In this review, “natural products” were defined as interventions of natural origin, including botanical decoctions, coded herbal drugs, prebiotics/fibers, and natural compound interventions (such as polyphenols). Probiotics, synthetic derivatives, and non-natural pharmacological compounds were excluded. Inclusion criteria were clinical trials (non-randomized clinical trials; randomized, double-blind, placebo-controlled trials; observational studies; case-control studies; single-group studies; retrospective studies; single-arm pre-post studies) published in English between 2013 and 2023, covering a 10-year period. Studies were excluded if they were published outside the specified period, in languages other than English, or if they reported *in vitro* or *in vivo* experiments. Systematic reviews, book chapters, and conference abstracts were also excluded (Figure 2).

### Data extraction

Data extraction was independently performed by at least three authors, with D.M.-F. consulted to resolve any discrepancies. Information from each included study was systematically collected according to predefined criteria: study design, year, study duration, total number of patients, patient age, patient sex, antineoplastic regimen, gastrointestinal problems associated with diarrhea, treatment formulation, treatment regimen, and main outcomes. All extracted data were compiled and analyzed in strict accordance with the inclusion and exclusion criteria defined for this systematic review.

The botanical nomenclature of all plant species included in this study was verified against the World Flora Online (WFO) and the Medicinal Plant Names Services (MPNS) databases to ensure the use of currently accepted scientific names, correct authorship attribution, and accurate family-level classification. Plant names reported in the original publications were cross-checked against these authoritative sources, and a harmonized nomenclature was consistently applied throughout the manuscript following the format: Genus species Authority [Family; pharmacopoeial botanical drug name], as presented in Supplementary Table 2. For species lacking an official pharmacopoeial botanical drug name or monograph, only the accepted scientific name and family classification were retained. In addition, the chemical and taxonomic characterization of the plant extract was performed in accordance with the ConPhYMP guidelines. The completed ConPhYMP checklist is provided in the Supplementary Material (Supplementary Table 1).

### Risk of bias assessment

Risk of bias was assessed using the RoB 2.0 tool for randomized trials, ROBINS-I for non-randomized studies, and the National Institutes of Health (NIH) National Heart, Lung, and Blood Institute (NHLBI) quality assessment tools for single-arm pre-post and case-control study designs. This assessment was performed by three authors, with D.M.-F. responsible for reviewing any differing data. The assessment included the randomization process, deviations from intended interventions, missing outcome data, measurement of the outcome, and selection of the reported result. Risk of bias was classified as ‘low risk,’ ‘high risk,’ or ‘some concerns’ for randomized trials (Hooijmans et al., 2014). For non-randomized studies, the ROBINS-I tool was used, categorizing risk as ‘low,’ ‘moderate,’ ‘serious,’ or ‘critical’ risk of bias (Sterne et al., 2016). For the NIH tool, each item was evaluated as ‘Yes,’ ‘No,’ ‘CD’ (cannot determine), ‘NA’ (not applicable), or ‘NR’ (not reported), with the overall quality of the study rated as ‘good,’ ‘fair,’ or ‘poor’ (NHLBI, 2021).

### Quality assessment of evidence

The certainty of the evidence was evaluated using a GRADE (Grading of Recommendations Assessment, Development and Evaluation) informed framework and was applied qualitatively due to the absence of meta-analysis. Outcomes of interest, along with their respective populations, interventions, and comparators, were defined *a priori*. Certainty ratings were initially based on study design, with randomized controlled trials considered high-certainty evidence and observational studies classified as low-certainty evidence. The evidence was then appraised across the five GRADE domains: risk of bias, inconsistency (considering both the direction and magnitude of effects), indirectness, imprecision, and publication bias. Downgrading for imprecision and publication bias was applied due to the lack of quantitative synthesis and the predominance of small sample sizes, both of which limited the strength of these assessments. Overall certainty was determined according to the cumulative downgrades and categorized as high, moderate, low, or very low. For observational studies, upgrading was considered when large effect sizes, evidence of a dose-response relationship, or situations in which plausible biases would likely attenuate the observed effect were identified.

### Statistical significance of reported outcomes

Statistical significance was systematically extracted from the included studies as reported by the original authors, including the statistical tests used and corresponding p-values to inform the interpretation of intervention effects.

### Subgroup analysis and mechanistic synthesis

An exploratory subgroup synthesis was conducted based on diarrhea etiology (oncological vs. non-oncological) and intervention class (botanical drug decoctions, *Boswellia serrata* Roxb [Burseraceae], prebiotics/fibers, proprietary multi-botanical drug formulations [e.g., Entoban® and Amoebex®], and polyphenol-based dietary interventions). This approach aimed to identify patterns in statistically significant outcomes, explore plausible mechanisms of action underlying the observed effects, and highlight areas where the low certainty of the available evidence warrants cautious interpretation.

### Safety considerations and herb-drug interaction assessment

A descriptive, safety-focused analysis was conducted to better contextualize the herbal interventions included in this review. The objective was to summarize proposed anti-diarrheal mechanisms, pharmacokinetic characteristics, potential herb-drug interactions, and key safety domains, with particular attention to oncology settings where polypharmacy is common. This analysis did not assess clinical efficacy and was not incorporated into the GRADE framework.

Data were drawn from preclinical research, human pharmacokinetic and safety studies, pharmacological reviews, regulatory documents, and authoritative monographs. For each intervention, the available information was synthesized narratively and presented in a dedicated table outlining proposed mechanisms of action, known or theoretical interactions, potential safety concerns, relevance to oncology, the level of pharmacological or toxicological evidence, and primary sources. The reported level of evidence refers exclusively to pharmacological or toxicological data and should not be interpreted as indicative of clinical effectiveness.

## Results

### Selection of studies

The initial search identified 732 articles. After removing duplicates, 728 were screened by title and abstract, resulting in 19 articles for full-text analysis. In parallel, a refined manual search, which involved analyzing reference lists of relevant systematic reviews and tracking citation using the same descriptors as the electronic search, identified 15 additional articles not captured by the initial databases. After a full-text review of the 34 papers, all 19 articles from the electronic database search were excluded for failing to strictly meet the inclusion criteria (e.g., unrelated outcomes or lack of a control group). In contrast, 10 of the 15 manually identified articles met all requirements and were retained for data extraction (Figure 2). This discrepancy may be due to the selected studies being published in journals with specific indexing or using herbal medicine terminology not fully mapped by MeSH terms.

The selected studies highlight key findings on the treatment of diarrhea associated with or without chemotherapy using natural products (Table 4). They also provide relevant details on study participants, natural product interventions, and study design, which are summarized in Table 5.

**TABLE 4:** Summary of key findings from selected studies investigating the effects of natural products on the management of diarrhea associated with or without chemotherapy.

Group	Study design	Year	Duration	Total patients (n)	Age	Gender	References
Chemotherapy-related diarrhea	Prospective comparative non-randomized clinical study, stratified by genotype	2017	June 2011 to February 2014	115	18–75 years old	-	Deng et al. (2017)
	Randomized double-blind placebo-controlled trial	2017	October 2015 to November 2016	20	24–84 years old (median: 57.55 years old)	Male (13) and female (7)	Sahebnasagh et al. (2017)
	Prospective, non-randomized observational study	2018	June 2014 and May 2017	27	45–72 years old (median: 62)	Male (25) and female (2)	Lu et al. (2018)
	Prospective randomized, double-blind, placebo controlled clinical trial	2020	13 months	42	18 and above	Male (27) and female (15)	Sahebnasagh et al. (2020)
	Randomized double-blind controlled trial	2021	-	30	18 and above	Male (8) and female (22)	Rosli et al. (2021)
Non-chemotherapy-induced diarrhea	Case control, double-blind, multicenter evaluation study	2016	-	184	15–45 years old	Male (103) and female (81)	Shah et al. (2016)
	Controlled, randomized and multicenter clinical trial study	2016	-	93	5–60 years old	Male and female	Shakeel et al. (2016)
	Single-group, retrospective evaluation	2020	June 2017 and May 2019	39	36.5 ± 11.8 years old (male) 32.8 ± 12.4 (female) years old	Male (22) and female (17)	Di et al. (2020)
	Non-randomized pre-post clinical study with internal control phases. Single-arm pre-post study	2020	May 2018 and January 2019	42	50 years old (mean)	Male (24%) and female (76%)	Ried et al. (2020)
	Randomized, double-blind, placebo-controlled clinical trial	2022	-	49	18–70 years old	Male (22) and female (27)	Giacosa et al. (2022)

**TABLE 5:** Overview of study characteristics.

Group	Antineoplastic regimen	Gi problem	Treatment formulation	Treatment regimen	Main outcomes	Adverse events	References
Antineoplastic-related diarrhea	5-Fluorouracil plus l-leucovorin (FOLFIRI)	Nausea, vomiting and diarrhea	*Shengjiang Xiexin* decoction (SXD) (*Zingiber officinale, Glycyrrhiza glabra, Codonopsis pilosula, Zingiber officinale, Astragalus mongholicus, Pinellia ternata, Coptis chinensis, and Ziziphus jujuba*)	100 mL orally, twice a day, (1 day before chemotherapy, and continue until 6 days after chemotherapy)	↓ nausea, vomiting and diarrhea	Not reported	Deng et al. (2017)
	External-beam radiation therapy	Proctitis	*Aloe vera* 3% ointment	1 g Oitment applied rectally, twice daily (4 weeks)	↓ symptom index for diarrhea, fecal urgency, overall clinical presentation and RTOG total ↑ lifestyle score	No	Sahebnasagh et al. (2017)
	CPT-11	Diarrhea	*Banxia Xiexin* decoction *(Pinellia ternata, Scutellaria baicalensis, Zingiber officinale, Panax ginseng, Coptis chinensis, Ziziphus jujuba,* and *Glycyrrhiza glabra)*	Orally twice a day (5 consecutive days, starting 1 day before the second cycle of chemotherapy) *No dose information	↓ diarrhea	Not reported	Lu et al. (2018)
	External beam radiotherapy	Proctitis	*Aloe vera* 3% ointment	1 g Oitment applied rectally, twice daily (6 weeks)	↓ symptom index for diarrhea, fecal urgency, rectal bleeding ↑ lifestyle score, depression status in HAD scale ↓ systemic inflammation (C-reactive protein)	No	Sahebnasagh et al. (2020)
	Pelvic radiation	Diarrhea	Gucil™ - *Cyamopsis tetragonoloba* seeds (Partially hydrolyzed guar gum - PHGG)	10 g orally (dissolved in 100 mL of water), twice a day, (28 days: 14 days before and 14 days during pelvic radiation therapy)	↓ diarrhea frequency ↑ of types 4 and 5 of stool types by BSS score ↑ bifidobacterium count	Not reported	Rosli et al. (2021)
Non- antineoplastic-induced diarrhea	-	Amoebic dysentery	Amoebex	400 mg orally (2 tablets), after meal, Three times a day *No information on period/time of treatment	↓ stool with blood/mucus, loose stool, diarrhea ↓ of abdominal pain, nausea, vomiting ↑ eradication rate of amoebiasis	No	Shah et al. (2016)
	-	Chronic diarrhea	Entoban (*Myrtus communis, Butea frondosa, Aegle marmelos, Berberis aristata, Holarrhena pubescens, and Quercus infectoria*)	400 mg orally, once every 8 h (5 days)	↓ diarrhea symptoms, abdominal pain, anorexia, flatulence, nausea, vomiting, rectal urgency, incontinence and bloating ↑ intention-to-treat	Metallic taste, anorexia, and headache	Shakeel et al. (2016)
	-	Diarrhea or diarrhea type irritable bowel syndrome	*Berberis aristata* (Dibiesse®) each tablet contains 250 mg of berberine (from *Berberis aristata* extract, standardized to not less than 97% of pure berberine); melatonin (0.5 mg); and depolymerized guar gum (DGG, 750 mg)	250 mg orally Every 12 h (90 days)	↓ number of stool and diarrhea symptoms ↑ of types 3 and 4 of stool types by BSS score	No	Di et al. (2020)
	-	Moderate GI disturbances of the upper and/or lower GI tract (including Regurgitationnausea and diarrhea)	NC gut relief *(Curcuma longa*, glutamine, quercetin, glucosamine hydrochloride, *Aloe vera, Ulmus rubra*, *Cyamopsis tetragonoloba*, pectin, peppermint oil, and dibasic sodium phosphate (powdered supplement)	5 g/day orally (first month) 10 g/day orally (second month) 0, 5, or 10 g/day orally (third month) *Mixed in water and/or food	↑ of type 4 of stool type by BSS score ↓ indigestion, heartburn, nausea, constipation, diarrhea, abdominal pain, bloating, troublesome flatulence, intestinal permeability ↑ leeds dyspepsia questionnaire, GERD-Q, GERD-QoL and GERD-HRQL for overall quality of life ↑ gut microbiome	No	Ried et al. (2020)
​	-	Acute diarrhea	Lecithin-based delivery form of *Boswellia serrata*	250 mg orally, twice a day, (5 days)	↓ number of stools ↓ of abdominal pain, nausea **↑** GAE	Not reported	Giacosa et al. (2022)

Abbreviations: ↓ decrease, ↑ improvement; BSS, bristol stool scale; FACT-G, The Functional Assessment of Cancer Therapy-General; GAE, global assessment of efficacy; GERD-HRQL, Gastroesophageal Reflux Disease - Health-Related Quality of Life; GERD-Q, gastroesophageal reflux disease questionnaire; GERD-QoL, Gastroesophageal Reflux Disease - Quality of Life; HAD, Hospital Anxiety-Depression Scale; HiPP, Hipp GmbH and Co. Vertrieb KG; PHGG, partially hydrolyzed guar gum; RTOG, radiation therapy oncology group criteria.

### Main findings

Of the 10 articles included in this review, 4 (40%) evaluated natural product preparations in the context of oncology-induced diarrhea, and 1 (10%) investigated a commercial natural product preparation for chemotherapy-induced diarrhea. Two (20%) investigated chemotherapy-induced diarrhea, 3 (30%) investigated radiation-induced diarrhea, and 5 (50%) investigated natural products not associated with chemotherapy- or radiation-induced diarrhea. All studies analyzed parameters related to gastrointestinal changes, including nausea, vomiting, appetite, diarrhea, pain, presence of blood or mucus in stool, fecal urgency, rectal bleeding, and daily stool frequency. Of the 10 articles, 3 (30%) used the Bristol Stool Scale to assess stool consistency, and 4 (40%) assessed quality of life using both standardized, validated instruments and study-adapted questionnaires. The results are described below as primary outcomes (e.g., clinical control of diarrhea) and secondary outcomes (e.g., associated gastrointestinal symptoms and quality of life).

### Natural product preparations for the treatment of antineoplastic-associated diarrhea

Deng et al. (2017) conducted a nonrandomized clinical trial to evaluate the effects of the traditional botanical drug formulation Shengjiang Xiexin Decoction (SXD) on hematologic and gastrointestinal toxicities induced by irinotecan in patients receiving the FOLFIRI chemotherapy regimen. During chemotherapy, patients were specifically asked about diarrhea, nausea, vomiting, and appetite using a non-standardized questionnaire. The results showed that SXD alleviated neutropenia, diarrhea, nausea, and vomiting. Antitumor efficacy was assessed according to the Response Evaluation Criteria in Solid Tumors (RECIST). Of the 115 patients included, 86 showed a response: 11 had a partial response, 55 had stable disease, and 20 had progressive disease. Importantly, SXD administration did not compromise the antitumor efficacy of chemotherapy. The study did not include detailed safety data on adverse events specifically related to SXD. In addition, patients’ quality of life was not assessed using standardized questionnaires. Although participants were screened for UGT1A128 and UGT1A16 polymorphisms prior to chemotherapy, the study did not further explore how these genetic variants influenced outcomes.

Sahebnasagh et al. (2017) conducted a randomized, double-blind, placebo-controlled study to investigate the effects of a topical ointment containing a 3% *Aloe vera* preparation (pure spray-dried Aloe vera powder). This product consists of the inner gel from the plant), on radiotherapy-induced proctitis in patients with malignant pelvic tumors who developed acute radiation proctitis after external beam radiotherapy. Twenty patients were randomly assigned to receive either 3% *Aloe vera* or placebo ointment, applied rectally at a dose of 1 g twice daily for 4 weeks; all patients also received sulfasalazine (500 mg, four times daily). The study assessed the severity of proctitis-related symptoms, including diarrhea, fecal urgency, and overall clinical presentation, using a symptom severity scale from 0 (not present) to 4 (causing significant discomfort). The reduction in symptoms assessed by the total RTOG was significant (p = 0.007) after 4 weeks of treatment with *Aloe vera*, compared to the placebo group. This reduction was particularly evident in symptoms associated with diarrhea (p = 0.015) and proctitis (p = 0.037). Improvement in lifestyle and daily functioning was also evaluated using a questionnaire ranging from 0 (no impairment of daily activities) to 4 (fear of leaving the house), with greater improvement observed in the *Aloe vera* group. The final lifestyle impact score in the treated group decreased significantly (from 1.1 to 0.33) compared to the placebo group (p = 0.004), directly reflecting the absence of symptoms that interfered with the daily activities of treated patients. The ointment was generally well tolerated, and no significant adverse events were reported during the treatment period. The mean age of patients in the *Aloe vera*-treated group (n = 19) was 61.5 ± 13.1 years, whereas those in the placebo group had a mean age of 63.3 ± 11.8 years. The treated group consisted of 11 males and eight females, while the placebo group comprised 16 males and seven females. The combined mean age across both groups was 62.5 ± 12.3 years, with a gender distribution of 27 males (34.3%) and 15 females (35.7%).

Sahebnasagh et al. (2020) expanded their earlier randomized controlled trial by further examining the effects of 3% *Aloe vera* ointment on radiotherapy-induced proctitis in patients receiving external beam radiotherapy for malignant pelvic tumors. This prospective, randomized, double-blind, placebo-controlled study included 42 patients who were randomly assigned to receive either 3% *Aloe vera* ointment or placebo, administered rectally with an applicator twice daily (1 g per application) for 6 weeks starting on the first day of radiotherapy. Treatment adherence was monitored using patient diaries. The study assessed symptom severity, including diarrhea, urge to defecate, rectal bleeding, and overall symptom index, using a scale from 0 (absent) to 4 (causing significant discomfort). Treatment with *Aloe vera* reduced symptom severity compared with placebo and improved lifestyle scores and depression status, as measured by the Hospital Anxiety and Depression Scale. Systemic inflammation was also evaluated, with aloe vera treatment associated with lower C-reactive protein levels during the 6-week follow-up. Overall symptom improvement was significant in patients treated with Aloe vera (p < 0.001), particularly reflected in the reduction of diarrhea (p < 0.001), rectal bleeding (p < 0.001), and fecal urgency (p = 0.001). Patients in the treatment group also demonstrated a significant improvement in lifestyle scores (p < 0.001), as well as a significant reduction in depression status according to the HAD scale (p = 0.049). Furthermore, treated patients exhibited a significant decrease in C-reactive protein levels (p = 0.009) compared to the placebo group. As in the previous study, the intervention was well tolerated, and no significant adverse events were reported.

Lu et al. (2018) investigated the effect of the traditional multi-botanical drug formulation *Banxia Xiexin* Decoction on irinotecan-induced diarrhea in a non-randomized observational study of 27 Chinese patients with recurrent small cell lung cancer receiving chemotherapy. The traditional multi-botanical drug formulation consisted of *Pinellia ternata* (Thunb.) Breit [Araceae; Pinelliae Rhizoma], *Scutellaria baicalensis Georgi* [Lamiaceae; Scutellariae Radix], *Zingiber officinale Roscoe* [Zingiberaceae; Zingiberis Rhizoma], *Panax ginseng* C.A. Mey [Araliaceae; Ginseng Radix et Rhizoma], *Coptis chinensis* Franch [Ranunculaceae; Coptidis Rhizoma], *Ziziphus jujuba* Mill [Rhamnaceae; Ziziphi Fructus], and *Glycyrrhiza glabra* L [Fabaceae; Glycyrrhizae Radix et Rhizoma] was administered orally as a liquid decoction for five consecutive days, beginning 1 day before the second chemotherapy cycle, with dosage adjustments based on the occurrence and severity of diarrhea. Symptomatic treatments such as loperamide were allowed, either alone during the first cycle or in combination with the decoction during the second cycle if diarrhea occurred. Delayed diarrhea was assessed using the National Cancer Institute Common Toxicity Criteria for Adverse Events (version 4.0), and treatment with *Banxia Xiexin* Decoction was reported to effectively prevent or control symptoms. Tumor response was also reported: no complete responses were observed, six patients had a partial response, and ten had stable disease. All patients were genotyped for UGT1A1*28, UGT1A1*6, ABCB1*2, and SLCO1B1*15 polymorphisms; wild-type UGT1A1*28 was associated with lower toxicity, while UGT1A1*6 mutations and specific ABCB1*2 and SLCO1B1*15 variants were associated with higher toxicity, although diarrhea was predominantly mild (grade 1–2) due to the low irinotecan dose. The study did not report detailed treatment-related adverse events or assess quality of life using standardized instruments.

**FIGURE 3:**
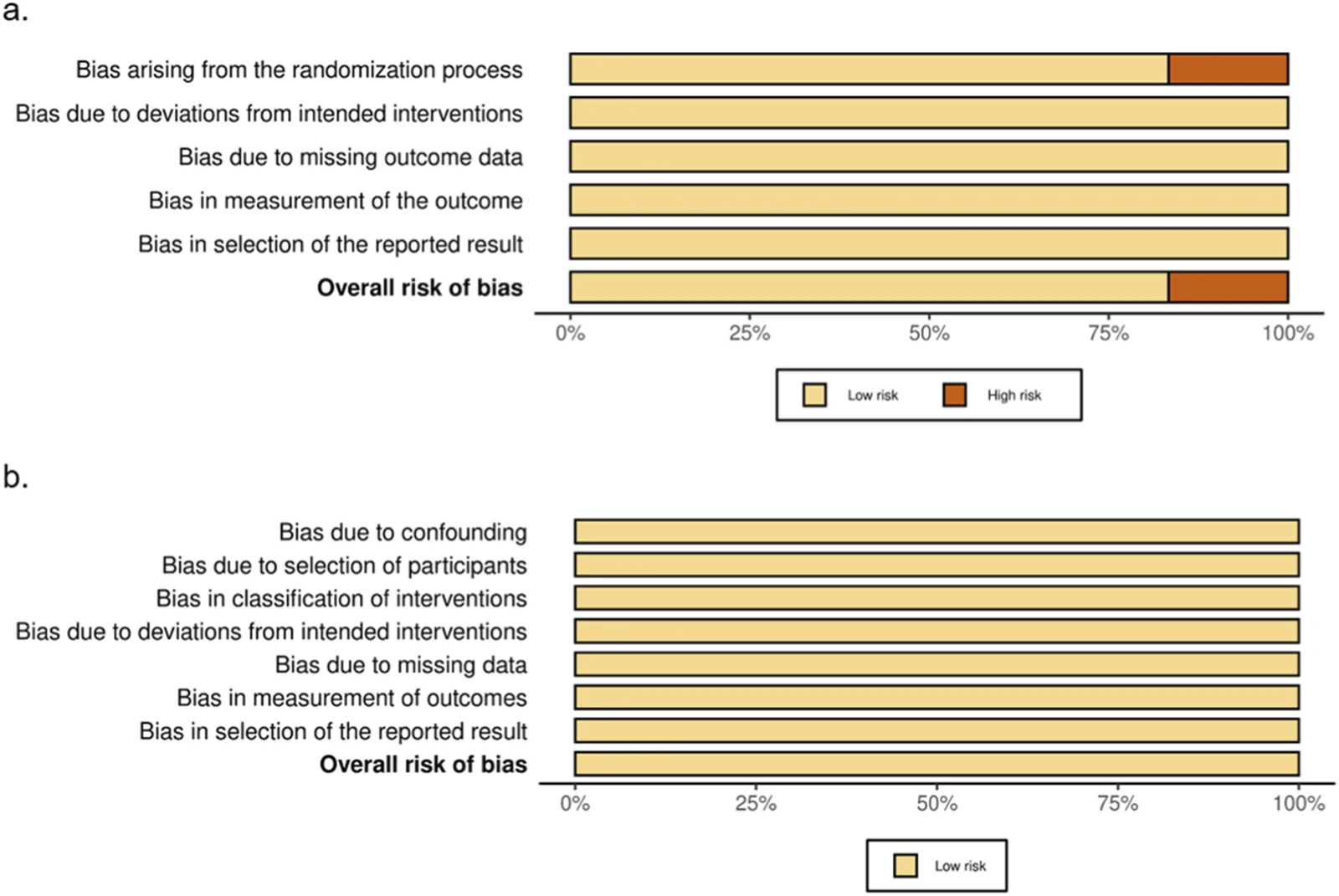
Assessment of Methodological Quality and Risk of Bias. The percentage of articles in each category is shown in color and in bars, with light orange representing low-risk studies and orange representing high-risk studies. **(a)** For randomized trials using RoB 2.0; **(b)** for non-randomized studies using ROBINS-I.

Rosli et al. (2021) conducted a double-blind, randomized controlled trial to investigate the effect of Gucil™, a partially hydrolyzed guar gum (PHGG) preparation derived from the seed endosperm of *Cyamopsis tetragonoloba* (L.) Taub [Fabaceae], on radiation-induced diarrhea in patients undergoing pelvic irradiation for endometrial, cervical, colon, rectal, or prostate cancer. Thirty patients were randomized to receive either 10 g PHGG or maltodextrin (control), dissolved in 100 mL of water and administered twice daily for 28 days, starting 14 days before and continuing throughout pelvic radiotherapy. The treated group (IG) presented a mean age of 57 ± 11.1 years, consisting of five males (35.7%) and nine females (64.3%). The control group presented a mean age of 55 ± 10.7 years, comprising three males (18.8%) and 13 females (81.3%). The combined mean age of patients across both groups was 55.9 ± 10.8 years, with a gender distribution of eight males (26.7%) and 22 females (73.3%). Patients requiring antibiotics or antidiarrheal drugs received appropriate medication and were excluded from the study. The incidence and severity of diarrhea were assessed using the National Cancer Institute Common Terminology Criteria and the Bristol Stool Chart. PHGG supplementation reduced diarrhea incidence at the end of radiotherapy (day 45) following 2 weeks of supplementation. The intervention was also associated with increased *Bifidobacteria* levels. The relative abundance of *Bifidobacterium spp*. Increased during the first 14 weeks of treatment in the treated group (0.046) compared to the control group (0.019) (Figure 3), followed by a subsequent decline in weeks 28 and 45, which was associated with radiotherapy. A significant temporal difference was observed in relation to PHGG supplementation (p = 0.026), despite the absence of significant differences in the total bacterial count at the end of treatment. Functional scales, symptom scales, and global health status were evaluated using the EORTC QLQ-C30 (version 3.0), although no significant differences were observed between the treatment and control groups. The study did not report detailed safety data on adverse events related to the intervention.

### Natural product preparations for the treatment of diarrhea unrelated to antineoplastic treatment

Shah et al. (2016) conducted a randomized, double-blind clinical trial to evaluate the efficacy, safety, and associated effects of the multi-botanical drug formulation Amoebex (400 mg tablets), whose principal botanical ingredient is *Holarrhena pubescens* Wall. Ex G. Don [Apocynaceae], compared with conventional treatment using metronidazole (400 mg) in 184 patients diagnosed with amebiasis. Among the patients allocated to the group treated with Amoebex, the mean age was 28.14 years for males (n = 55) and 27.82 years for females (n = 38). In the control group, the mean age was 27.95 years for males (n = 48) and 29.49 years for females (n = 43) (Table 2). The combined mean age across both groups was 27.3 ± 7.16 years, with gender distribution comprising 103 females (56%) and 81 males (44%).

**TABLE 2:** Quality assessment of case-control studies.

Criteria	Yes	No	Other (CD, NR, NA)
1. Was the research question or objective in this paper clearly stated and appropriate?	X	​	​
2. Was the study population clearly specified and defined?	X	​	​
3. Did the authors include a sample size justification?	​	​	NR
4. Were controls selected or recruited from the same or similar population that gave rise to the cases (including the same timeframe)?	X	​	​
5. Were the definitions, inclusion and exclusion criteria, algorithms or processes used to identify or select cases and controls valid, reliable, and implemented consistently across all study participants?	X	​	​
6. Were the cases clearly defined and differentiated from controls?	X	​	​
7. If less than 100 percent of eligible cases and/or controls were selected for the study, were the cases and/or controls randomly selected from those eligible?	X	​	​
8. Was there use of concurrent controls?	​	​	NR
9. Were the investigators able to confirm that the exposure/risk occurred prior to the development of the condition or event that defined a participant as a case?	X	​	​
10. Were the measures of exposure/risk clearly defined, valid, reliable, and implemented consistently (including the same time period) across all study participants?	X	​	​
11. Were the assessors of exposure/risk blinded to the case or control status of participants?	X	​	​
12. Were key potential confounding variables measured and adjusted statistically in the analyses? If matching was used, did the investigators account for matching during study analysis?	X	​	​

Abbreviations CD, cannot determine; NA, not applicable; NR, not reported.

Parasitological outcomes were assessed by stool analysis after treatment, confirming eradication of *Entamoeba histolytica* in 70.96% of patients treated with Amoebex and 57.14% in the metronidazole group. Clinical outcomes were evaluated using a Likert scale and included diarrhea, abdominal pain, and the presence of blood or mucus in the stool. Treatment with Amoebex was associated with greater improvement in clinical symptoms, with abdominal pain resolving in more than 93% of patients and diarrhea decreasing in 76% of cases, compared with 20% in the metronidazole group. The frequency and severity of nausea, vomiting, and mucus in the stool were also significantly reduced in the Amoebex group. No adverse effects were reported during the study.

Shakeel et al. (2016) conducted a randomized, controlled, multicenter clinical trial to evaluate the efficacy, safety, and clinical effects of Entoban, a multi-botanical drug formulation manufactured by *Herbion Pakistan* (Pvt.) and administered as 400 mg capsules, compared with metronidazole for the treatment of chronic diarrhea in 150 patients. Overall, 95 males and 55 females were included in the control and test group, respectively. The age range for the test and control groups was 25 ± 11.86 and 23 ± 13.76 years, respectively. Outcomes were assessed over a 4-week period using a 0–3 scoring system and included daily stool frequency, abdominal discomfort, stool consistency, sensation of incomplete evacuation, and flatulence. Treatment with Entoban resulted in a significant reduction in bowel movement frequency compared with metronidazole at week four, along with greater and more sustained improvements in overall symptom severity. Complete clinical improvement was observed in 84.78% of patients in the Entoban group compared with 78.72% in the control group. Among complete responders, the median total symptom score decreased to 4 after 4 weeks. Adverse effects were reported less frequently in the Entoban group than in the metronidazole group, with the latter commonly associated with anorexia, metallic taste, dizziness, and vomiting. These findings suggest that Entoban may be a viable botanical drug alternative for long-term symptomatic management of chronic diarrhea, particularly in patients for whom conventional therapy is limited by adverse effects.

Di et al., 2020 conducted a retrospective single-group analysis to evaluate the effects of 3 months of supplementation with Dibiesse® (250 mg tablets), a formulation containing *Berberis aristata* DC [Berberidaceae] extract standardized to ≥97% berberine (250 mg per tablet), melatonin (0.5 mg), and depolymerized guar gum (DGG, 750 mg), in 39 patients diagnosed with functional gastrointestinal disorders, including irritable bowel syndrome and functional diarrhea. After supplementation, patients showed significant improvement in gastrointestinal symptoms such as diarrhea, abdominal pain, bloating, altered bowel habits, and flatulence, as assessed using a symptom severity scale ranging from 0 to four points. After 3 months of therapy, patients in the functional diarrhea (FD) group demonstrated a significant improvement in stool types 3 (n = 4, p < 0.01) and 4 (n = 8, p < 0.001), according to the Bristol Stool Scale. In the irritable bowel syndrome with diarrhea (IBS-D) group, patients also reported significant improvement in stool type 3 (n = 5, p < 0.01) and type 4 (n = 4, p < 0.01). The intervention was generally well tolerated; the most frequently reported adverse effect was flatulence, while two patients reported skin rash and one patient with functional diarrhea reported stomach burning. Overall, the findings support the potential role of *B. aristata* extract supplementation as an adjunctive treatment for functional gastrointestinal disorders, including diarrhea.

Furthermore, it is important to emphasize that the gastrointestinal effects reported for depolymerized guar gum (DGG) in the formulation evaluated by Di et al., 2020 may differ from those observed for partially hydrolyzed guar gum (PHGG) in the study by Rosli et al. (2021). Although both ingredients are derived from guar gum obtained from *C. tetragonoloba* (L.) Taub [Fabaceae], PHGG is a specific, standardized form of depolymerized guar gum with well-documented physiological and prebiotic properties (Yoon et al., 2008). In contrast, DGG is a broader term that may refer to preparations with different degrees of depolymerization and distinct physicochemical characteristics. Consequently, caution is warranted when extrapolating findings obtained with PHGG to other DGG preparations.

The beneficial effects reported for PHGG likely reflect not only its physicochemical properties as a soluble dietary fiber but also its selective utilization by gut microorganisms, consistent with the current definition of a prebiotic (Gibson et al., 2017). By comparison, although Di et al., 2020 reported favorable clinical outcomes with DGG supplementation, the study did not evaluate microbial selectivity or prebiotic activity. Therefore, it remains unclear whether the observed effects were mediated primarily by the physicochemical properties of the fiber, by modulation of the gut microbiota, or by a combination of both mechanisms.

In a single-arm pre-post study, Ried et al. (2020) evaluated the effectiveness of the Nutrition Care Gut Relief powdered formula (5-10 g), containing *Curcuma longa*, *Aloe vera*, *Ulmus rubra* Muhl [Ulmaceae; Ulmi rubrae cortex], guar gum derived from *C. tetragonoloba* (L.) Taub [Fabaceae], peppermint oil, and glutamine, in 43 participants with gastrointestinal complaints, including indigestion, diarrhea, and constipation.

In individuals with irritable bowel syndrome, particularly the diarrhea-predominant subtype, use of the NC Gut Relief Formula was associated with marked improvement in gastrointestinal symptoms. NC Gut Relief Formula significantly reduced the number of daily bowel movements, improving stool frequency and regularity toward the ideal range of 1–2 times per day. The proportion of participants within this range increased from 58% at baseline to 79% after the 12-week intervention. Stool consistency also improved toward the ideal type 4 on the Bristol Stool Scale. Compared to baseline, significant reductions in abdominal pain, bloating, and urinary urgency were observed, with symptom scores on a 1–10 pain scale reaching statistical significance after 12 weeks of intervention. Improvements were also noted in constipation, diarrhea, and flatulence in 40%, 43%, and 58% of participants, respectively, according to the Birmingham Score. Quality of life, assessed using the GERD-QoL questionnaire, improved by 82% across domains including emotional wellbeing, sleep, energy levels, diet, and social interactions. Measures of intestinal permeability showed elevated lactulose and mannitol levels at baseline, indicative of increased mucosal pore size, which decreased by 59% and 27%, respectively, following supplementation. The intervention was also associated with favorable modulation of the gut microbiota, with increases in non-pathogenic bacteria such as *Lactobacilli* (29%), *Faecalibacterium prausnitzii* (27%), and Clostridia species (36%). No adverse effects were reported during the study period.

Giacosa et al. (2022) evaluated the effects of a lecithin-based formulation of *B. serrata* Roxb. Ex Colebr [Burseraceae] extract (250 mg tablets) in 49 patients with acute diarrhea in a randomized, double-blind, placebo-controlled clinical trial. Treatment with *B. serrata* extract was associated with faster resolution of diarrhea, with cessation occurring within 3 days compared to 5 days in the placebo group, and a higher rate of complete recovery after 5 days (95.8% *versus* 76%). The intervention was also associated with improvements in overall clinical status, as reflected by higher scores on the global assessment of efficacy (GAE) four-point scale, including reductions in nausea, vomiting, abdominal pain, and bloating. No relevant adverse effects were reported during the study period.

### Risk of bias within studies

The summary of the risk of bias assessment is shown in Figure 3; Table 1, 2. Of the 10 studies included in this review, six randomized controlled trials were evaluated with the RoB 2.0 tool, of which five were categorized as having a low risk of bias and one was classified as having a high risk of bias due to not meeting the randomization criteria (Figure 3a). Two studies were non-randomized and assessed using the ROBINS-I tool; both were also considered to have a low risk of bias (Figure 3b). Finally, the case-control study and the pre-post study were evaluated using the NIH quality assessment questionnaires, with both rated as ‘good’ quality (Tables 1, 2).

**TABLE 1:** Quality assessment tool for before-after (Pre-Post) Studies with no control group.

Criteria	Yes	No	Other (CD, NR, NA)*
1. Was the study question or objective clearly stated?	x	​	​
2. Were eligibility/selection criteria for the study population prespecified and clearly described?	x	​	​
3. Were the participants in the study representative of those who would be eligible for the test/service/intervention in the general or clinical population of interest?	x	​	​
4. Were all eligible participants that met the prespecified entry criteria enrolled?	x	​	​
5. Was the sample size sufficiently large to provide confidence in the findings?	x	​	​
6. Was the test/service/intervention clearly described and delivered consistently across the study population?	x	​	​
7. Were the outcome measures prespecified, clearly defined, valid, reliable, and assessed consistently across all study participants?	x	​	​
8. Were the people assessing the outcomes blinded to the participants’ exposures/interventions?	​	x	​
9. Was the loss to follow-up after baseline 20% or less? Were those lost to follow-up accounted for in the analysis?	x	​	​
10. Did the statistical methods examine changes in outcome measures from before to after the intervention? Were statistical tests done that provided p values for the pre-to-post changes?	x	​	​
11. Were outcome measures of interest taken multiple times before the intervention and multiple times after the intervention (i.e., did they use an interrupted time-series design)?	x	​	​
12. If the intervention was conducted at a group level (e.g., a whole hospital, a community, etc.) did the statistical analysis take into account the use of individual-level data to determine effects at the group level?	​	​	CD

Abbreviations CD, cannot determine; NA, not applicable; NR, not reported.

### Summary of certainty of evidence (GRADE)

Certainty of evidence is reported in Table 3. Deng et al. (2017) reported reductions in irinotecan-related toxicities with *Shengjiang Xiexin Decoction* (SXD) in genetically susceptible patients; however, the lack of randomization and robust statistical analyses led to the certainty of evidence being downgraded from low to very low.

**TABLE 3:** Certainty of evidence (GRADE) for anti-diarrheal effects in oncological and non-oncological clinical settings.

Group	Study design	Intervention	Comparator	Primary outcome	Secondary outcome	Magnitude of the effect	Risk of bias	Direct evidence	Imprecision	Publication bias	Suggested grade	References
Chemotherapy-related diarrhea	Prospective comparative non-randomized clinical study, stratified by genotype	*Shengjiang Xiexin* decoction (SXD)	Comparison between patients with different genotypes (high-risk vs. wild type); this was not a formal placebo group	Incidence of diarrhea (grades I-II) and gastrointestinal toxicity associated with irinotecan	Neutropenia, nausea, vomiting, anorexia, infection; clinical response to treatment	Lower proportion of mild or moderate diarrhea and toxicities in patients treated with SXD	High (lack of randomization and placebo group, possible confounding factors)	Reduction of hematological and gastrointestinal toxicities in patients with high genetic risk	Serious diarrhea events were few and lacked robust statistical analysis	Possible (area with few controlled clinical studies)	Low to very Low	Deng et al. (2017)
	Randomized, double-blind, placebo-controlled clinical trial	*Aloe vera* 3%	Placebo (ointment without *Aloe vera*)	Reduction in the severity of proctitis symptoms, including diarrhea	Fecal urgency; total symptom index; radiation therapy oncology group (RTOG) criteria; impact on quality of life	The median diarrhea score decreased from approximately 0.67 to approximately 0.11 points during the intervention weeks	Moderate (small sample size; potential variation in subjective symptom assessment)	Favorable to *Aloe vera.* Significant reduction in diarrhea severity and overall symptom index (p < 0.05)	Moderate (small sample size, estimates with variability)	Possible (field with few published clinical trials)	High to Low	Sahebnasagh et al. (2017)
	Prospective, non-randomized observational study	*Banxia Xiexin* decoction	Absent (observational; intra-patient or historical comparison)	Prevention and control of late-onset irinotecan-induced diarrhea	General toxicity, tumor response, and survival (exploratory)	Low to moderate (based on a limited number of cases)	High (absence of randomization, blinding, and control group)	Serious (very specific population; nonstandardized formula)	Serious (very small number; few events)	Serious concerns (single study; positive results only; complementary medicine field)	Low	Lu et al. (2018)
	Prospective randomized, double-blind, placebo controlled clinical trial	*Aloe vera* 3%	Placebo (inactive ointment)	Incidence and severity of acute proctitis symptoms, including diarrhea	Rectal bleeding, fecal urgency, quality of life, CRP (systemic inflammation)	There was a significant reduction in the occurrence of diarrhea symptoms over 6 weeks of RT compared to placebo	Moderate (strong concept, but limited sample size and assessment of subjective symptoms)	Significantly favors *Aloe vera* in reducing diarrhea and other symptoms (p < 0.001)	Moderate (small N; estimates depend on symptom assessment and patient diaries)	Possible (a few trials using *Aloe vera* to predict proctitis)	High to Low	Sahebnasagh et al. (2020)
​	Randomized double-blind controlled trial	Gucil™ - PHGG)	Placebo (maltodextrin)	Frequency of diarrhea during and after radiation	Bifidobacterium counts in fecal microbiota, nutritional status, quality of life	There was a significant reduction in the occurrence of diarrhea symptoms over 6 weeks of RT compared to placebo	Moderate (randomization and blinding are well described; small sample size)	There was a trend toward reduced diarrhea frequency at the end of follow-up (day 45) in the PHGG group	Moderate to serious (small sample size; temporal variability of outcomes)	0 (no clear signal with this single study)	High to moderate	Rosli et al. (2021)
Non-chemotherapy-induced diarrhea	Case control, double-blind, multicenter evaluation study	Amoebex	Metronidazole (400 mg)	Clinical cure and eradication of infection (symptoms of dysentery, bloody or mucous diarrhea)	Decreased stool with blood or mucus, loose stool, and diarrhea. Decreased abdominal pain, nausea, vomiting	Significantly superior overall efficacy (p < 0.03) with differences in sign and symptom variables (p < 0.0357)	Moderate (randomization noted; protocol deviations and blinding not fully detailed; methods not completely transparent)	Favorable to amoebex (greater efficacy observed compared to metronidazole)	Moderate (184 participants, reasonable but without detailed confidence intervals; limited effect estimates)	Possible (field with few comparative studies of herbal medicines)	High	Shah et al. (2016)
	Controlled, randomized and multicenter clinical trial study	Entoban	Metronidazole (400 mg)	Daily frequency of bowel movements	Abdominal pain, bloating, changes in stool consistency, and a feeling of incomplete bowel movement	84.78% with complete improvement in the entoban group compared to 78.72% in the control group; significant improvement	Moderate (randomization described, but blinding details are unclear; losses and dropouts are reported)	Comparable clinical efficacy was observed between entoban and metronidazole, with symptom reduction in both groups	Moderate (reasonable sample size, but no detailed confidence intervals are reported)	Possible (field with few comparative trials of herbal medicines)	High	Shakeel et al. (2016)
	Single-group, retrospective evaluation	*Berberis aristata* (Dibiesse®)	No	Reduction in diarrheal events and improvement in stool consistency	Number of bowel movements per week, adherence to treatment, tolerability	Moderate to significant reduction in diarrhea and frequency of bowel movements	High (nonrandomized, no control group)	Favorable (diarrhea reduced by approximately 50%–80% over time)	Serious (general population, self-reported)	Possible (nutritional supplements frequently reported retrospectively)	Low to very low	Di et al. (2020)
​	Non-randomized pre-post clinical study with internal control phases. Single-arm pre-post study	NC gut relief	Pre-intervention (internal control, pre-post study)	Reduction in the frequency and severity of gastrointestinal symptoms (including diarrhea)	Improved stool consistency (ideal type), reduced intestinal permeability, improved microbial profile, and improved quality of life	Reduction of gastrointestinal symptoms in 40%–60% of cases, including diarrhea observed in a subgroup	High (no external control group, nonrandomized, self-assessment)	Improvement in general gastrointestinal symptoms, including reduced diarrhea and better stool consistency	Serious (limited sample size, pre-post design study)	Possible (field with little robust evidence)	Low to very low	Ried et al. (2020)
	Randomized, double-blind, placebo-controlled clinical trial	Lecithin-based delivery form of *Boswellia serrata*	Placebo (capsules without the active ingredient, without *Boswellia serrata* extract in lecithin form)	Time to resolution of diarrhea	Reduction in bowel movements; improvement in symptoms and gastroesophageal reflux (GAE)	Small to moderate	Moderate (good practices, but limited sample size)	Favorable to the intervention (*Boswellia serrata* compared to placebo)	Small sample size, slight downgrade	Publication bias: serious concerns (few studies; only positive findings)	High to Low	Giacosa et al. (2022)

Two randomized, placebo-controlled trials by Sahebnasagh et al. (2017), Sahebnasagh et al. (2020) demonstrated significant reductions in diarrhea severity and incidence with topical 3% *Aloe vera* in patients undergoing pelvic radiotherapy. Despite favorable findings, small sample sizes and subjective symptom measures led to downgrading from high to low certainty evidence.

Lu et al. (2018) described symptom improvement with *Banxia Xiexin* Decoction in an observational cohort of patients with irinotecan-induced diarrhea, although methodological constraints, including the absence of a control group, resulted in downgrading from to low to very low certainty.

Rosli et al. (2020) found that partially hydrolyzed guar gum (PHGG) reduced diarrhea frequency and increased *Bifidobacterium* abundance in irradiated patients, supporting a microbiota-mediated effect certainty was rated from high to moderate due to sample size limitations.

Among non-oncological populations, Shah et al. (2016) reported superior clinical efficacy of Amoebex compared with metronidazole in amoebic dysentery, whereas Shakeel et al. (2016) observed comparable symptom improvement with Entoban but fewer adverse events. Both studies were rated as high certainty despite some methodological concerns. Conversely, observational and pre-post studies evaluating berberine-based supplementation (Di et al., 2020) and a multi-ingredient nutraceutical (Ried et al., 2020) reported symptomatic improvements but were downgraded to low or very low certainty due to design-related limitations.

Finally, a placebo-controlled randomized trial of lecithin-based *B. serrata* extract demonstrated a shorter time to recovery and improved stool-related outcomes; however, imprecision associated with the small sample warranted cautious interpretation, resulting in downgrading from high to low certainty.

### Summary of statistically significant findings

All included studies reported statistically significant differences between intervention and comparator groups for at least one diarrhea-related outcome. Evaluated endpoints included diarrhea incidence, frequency, severity, number of bowel movements, and overall clinical improvement.

In oncological settings, studies by Deng et al. (2017), Lu et al. (2018), Sahebnasagh et al. (2017), Sahebnasagh et al. (2020), and Rosli et al. (2020) demonstrated significant reductions in diarrhea frequency or severity associated with chemotherapy or radiotherapy (p < 0.05). Statistical analyses primarily used chi-square tests, independent t-tests, and repeated-measures analyses of variance.

Similarly, studies in non-oncological populations, including Shah et al. (2016), Shakeel et al. (2016), Di et al., 2020, Ried et al. (2020), and Giacosa et al. (2022), reported statistically significant improvements, particularly in diarrhea resolution and reductions in bowel frequency. However, in some cases, the specific statistical tests or exact p-values were not fully reported, limiting the transparency of the findings.

Overall, the statistical results suggest a favorable association between the evaluated interventions and improvements in diarrhea-related outcomes. Nevertheless, variability in statistical approaches and inconsistencies in p-value reporting warrant cautious interpretation (Table 6).

**TABLE 6:** Summary of reported statistical significance across included studies.

Group	Outcome analyzed	Comparator	Statistical test reported	Statistical difference	p-value	References
Chemotherapy-related diarrhea	Incidence of irinotecan-induced diarrhea	Intervention vs. control	Not reported	Yes	p < 0.05	Deng et al. (2017)
	Frequency of diarrhea	Intervention vs. placebo	Chi-square/t-test	Yes	p < 0.05	Sahebnasagh et al. (2017)
	Incidence and severity of irinotecan-induced diarrhea	Intervention vs. control	Chi-square	Yes	p < 0.05	Lu et al. (2018)
	Frequency of evacuations	Intervention vs. placebo	Independent t-test	Yes	p = 0.03	Sahebnasagh et al. (2020)
	Frequency of diarrhea during and after radiation	Intervention vs. control	Repeated-measures ANOVA	Yes	p < 0.05	Rosli et al. (2021)
Non-chemotherapy-induced diarrhea	Diarrhea resolution	Intervention vs. metronidazol	Chi-square	Yes	p < 0.05	Shah et al. (2016)
	Clinical improvement of chronic diarrhea	Intervention vs. placebo	Chi-square	Yes	p < 0.05	Shakeel et al. (2016)
	Frequency and consistency of stools	Intervention vs. control	t-test	Yes	p < 0.05	Di et al. (2020)
	Frequency of diarrhea	Intervention vs. placebo	Mixed-model ANOVA	Yes	p < 0.05	Ried et al. (2020)
	Frequency of diarrhea	Intervention vs. control	Chi-square/t-test	Yes	p < 0.05	​

### Subgroup analysis and mechanistic synthesis

The exploratory subgroup analysis (Table 7) categorized studies by diarrhea etiology (oncological vs. non-oncological) and intervention class (botanical decoctions, *B. serrata*, prebiotics/fibers, coded botanical drugs, polyphenol-based dietary interventions). In oncological studies, interventions such as botanical decoctions, prebiotics, fibers, and polyphenols were associated with statistically significant reductions in diarrhea frequency or severity. These effects were likely mediated by modulation of the gut microbiota, reinforcement of intestinal barrier integrity, and anti-inflammatory activity. However, the overall certainty of evidence for these outcomes was low to very low according to GRADE, reflecting methodological limitations, small sample sizes, and heterogeneity in outcomes.

**TABLE 7:** Subgroup analysis by diarrhea etiology and intervention class.

Intervention class	Etiology	Statistically significant outcome?	GRADE certainty	Hypothesized mechanism	References
Botanical decoctions (multi-herb)	Non-oncological	Yes	Low to very Low	Anti-inflammatory, barrier modulation	Deng et al. (2017)
	Oncological	Yes	Low to very Low	Anti-inflammatory, microbiota modulation	Lu et al. (2018)
Boswellia	Non-oncological	Yes	High to Low	Anti-inflammatory	Sahebnasagh et al. (2017)
	Non-oncological	Yes	High to Low	Anti-inflammatory	Sahebnasagh et al. (2020)
Prebiotics and fibers	Oncological	Yes	High to moderate	Microbiota modulation, SCFA production	Rosli et al., 2020
	Non-oncological	Yes	Low to very low	Microbiota modulation, barrier support	Di et al. (2020)
	Oncological	Yes	High to Low	Microbiota modulation, barrier support	Giacosa et al. (2022)
Coded herbal/nutraceutical	Non-oncological	Yes	High	Antimicrobial, anti-secretory	Shah et al. (2016)
	Non-oncological	Yes	High	Antimicrobial, anti-inflammatory	Shakeel et al. (2016)
Polyphenol-based dietary	Oncological	Yes	Low to very low	Antioxidant, anti-inflammatory, microbiota modulation	Ried et al. (2020)

Findings should be considered exploratory due to heterogeneity in study design, outcomes, and duration, as well as the limited number of studies per subgroup, which precluded quantitative synthesis. The results support hypothesis generation and may inform future etiology-specific randomized trials, identification of promising interventions, and testing of plausible mechanisms.

In non-oncological populations, interventions including botanical decoctions, *B. serrata* extracts, coded herbal drugs, and prebiotics/fibers also showed significant improvements. Proposed mechanisms included direct antimicrobial activity, anti-inflammatory effects, and microbiota modulation. As in the oncological subgroup, GRADE certainty was predominantly low or very low, and methodological limitations precluded strong clinical recommendations.

Overall, this subgroup synthesis highlights potential mechanistic differences by etiology and intervention class, while emphasizing the need for caution in interpretation given the limited quality and consistency of evidence. Importantly, statistical significance did not consistently translate into high certainty of evidence, reinforcing the need for cautious interpretation.

### Safety considerations and potential herb-drug interactions

Safety considerations proposed anti-diarrheal mechanisms, and potential herb-drug interactions were synthesized from primary clinical reports and authoritative external sources (Table 8). The evaluated botanical formulations and nutraceuticals exhibited heterogeneous but biologically plausible mechanistic profiles, including anti-inflammatory activity, modulation of gut motility, reinforcement of epithelial barrier function, and microbiota-mediated effects (Manach et al., 2005; Ammon, 2006; EFSA, 2010; Slavin, 2013).

**TABLE 8:** Safety considerations, proposed mechanisms, and potential herb-drug interactions of botanical interventions included in the review.

Intervention	Proposed anti-diarrheal mechanism	Known pharmacokinetic interactions	Known herb-drug interactions	Potential issue/safety domain	Potential relevance in oncology	Level of evidence	Primary clinical study
*Shengjiang Xiexin* decoction (SXD)	Anti-inflammatory effects; regulation of gut motility and epithelial barrier	Theoretical CYP3A4/CYP2C9 modulation by *Scutellaria baicalensis* and *Glycyrrhiza glabra*	Potential interaction with chemotherapeutics metabolized by CYP enzymes	Botanical variability; lack of standardization	High (chemotherapy-induced diarrhea)	Preclinical/traditional pharmacology^1^ ^,^ ^2^	Deng et al. (2017)
*Boswellia serrata* extract	Inhibition of leukotrienes and NF-κB-mediated inflammation	Possible CYP2C9 inhibition (theoretical); weak CYP2C9 inhibition (*in vitro*)	Possible interaction with anticoagulants and NSAIDs	Bleeding risk; GI discomfort	Moderate	Preclinical pharmacology^1^ ^,^ ^3^	Sahebnasagh et al. (2017)
*Boswellia serrata* extract	Reduction of inflammatory mediators; gut mucosal protection	Similar theoretical CYP2C9 interaction	Potential interaction with anticoagulants	Limited long-term safety data	Moderate	Preclinical/clinical pharmacology^1^ ^,^ ^2^	Sahebnasagh et al. (2020)
Partially hydrolyzed guar gum (PHGG)	Increased stool consistency; fermentation to SCFAs; microbiota modulation	No systemic absorption; no PK interactions	None reported	Generally safe; bloating at high doses	High (radiotherapy-associated diarrhea)	Human nutrition studies^4^ ^,^ ^5^	Rosli et al., 2020
*Banxia Xiexin* decoction	Anti-inflammatory effects; regulation of intestinal permeability	Possible CYP modulation by *Pinellia ternata* and *Scutellaria baicalensis* (theoretical)	Potential interaction with chemotherapy agents	Toxicity if improperly processed; formulation variability	High	Preclinical/traditional pharmacology^1^ ^,^ ^2^	Lu et al. (2018)
Polyphenol-rich dietary intervention	Antioxidant and anti-inflammatory effects; microbiota interaction	Modulation of CYP3A4, CYP1A2, and transporters (compound-dependent)	Possible interaction with irinotecan, tamoxifen (theoretical)	Variable bioavailability	Moderate	Nutritional pharmacology^1^ ^,^ ^6^	Ried et al. (2020)
Amoebex (coded botanical drug)	Antimicrobial and anti-secretory effects	Not characterized	Unknown	Poor pharmacokinetic and toxicological characterization	Low	Traditional medicine sources^7^ ^,^ ^9^	Shah et al. (2016)
Entoban (polyherbal formulation)	Antimicrobial and anti-inflammatory effects	Not characterized	Unknown	Limited independent safety evaluation	Low-moderate	Traditional medicine sources^1^ ^,^ ^2^	Shakeel et al. (2016)
Prebiotic formulation	Restoration of microbiota; barrier reinforcement	Not characterized	Rare infection risk in immunocompromised patients	Safety in severely immunosuppressed patients	High	Human clinical and safety studies^8^ ^,^ ^9^	Di et al. (2020)
Nutraceutical	Microbiota modulation; reduction of intestinal permeability	Not characterized	Minimal; context-dependent	Generally safe; immunosuppression caution	High	Human clinical studies^9^ ^,^ ^10^	Giacosa et al. (2022)

* Safety data, proposed mechanisms, and potential herb-drug interactions were synthesized from clinical studies and external pharmacological sources. Many interaction risks remain theoretical or mechanistically inferred; therefore, findings should be interpreted as risk-informed considerations rather than definitive clinical evidence. The absence of reported interactions should not be interpreted as evidence of safety.

1 Izzo AA, Ernst E. Interactions between herbal medicines and prescribed drugs: a systematic review. Drugs. 2009; 69 (13):1777–1798. Doi:10.2165/11317010-000000000-00000. Source supporting potential herb-drug interactions and cytochrome P450 modulation.

2 World Health Organization (WHO). WHO, Monographs on Selected Medicinal Plants. Geneva: WHO; 1999–2009. Source supporting safety considerations, toxicological profiles, and issues related to herbal standardization.

3 Ammon HP., Boswellic acids and their role in chronic inflammatory diseases. Planta Med. 2006; 72 (12):1,100–1,116. Doi:10.1055/s-2006-947227. Source supporting anti-inflammatory mechanisms and pharmacological activity of Boswellia serrata.

4 EFSA, panel on dietetic products, Nutrition and Allergies. Scientific opinion on dietary fibre. EFSA, Journal. 2010; 8 (3):1,462. Doi:10.2903/j.efsa.2010.1462. Source supporting safety and physiological effects of dietary fibers.

5 Slavin JL., Fiber and prebiotics: mechanisms and health benefits. Nutrients. 2013; 5 (4):1,417–1,435. Doi:10.3390/nu5031417. Source supporting microbiota-mediated mechanisms of fiber-based interventions.

6 Manach C, et al. Bioavailability and bioefficacy of polyphenols in humans. Am J Clin Nutr. 2005; 81 (1 Suppl):230S–242 S. doi:10.1093/ajcn/81.1.230S. source supporting pharmacokinetic variability and metabolic pathways of polyphenols.

7 World Health Organization (WHO). Guidelines on Safety Monitoring of Herbal Medicines in Pharmacovigilance Systems. Geneva: WHO; 2004. Source supporting the importance of pharmacovigilance and safety monitoring for herbal products.

8 Sanders ME, et al. Safety assessment of probiotics for human use. Gut Microbes. 2010; 1 (3):164–185. Doi:10.4161/gmic.1.3.12127. Source supporting safety considerations and infection risk in vulnerable populations.

9 FAO/WHO., Guidelines for the Evaluation of Probiotics in Food. london, Ontario; 2002. Source supporting regulatory and safety frameworks for probiotic use.

10 McFarland LV., Use of probiotics to correct dysbiosis: a systematic review. Ther Adv Gastroenterol. 2015; 7 (6):347–359. Doi:10.1177/1756283X14537189. Source supporting clinical safety and tolerability of probiotics.

Potential pharmacokinetic interactions were mainly theoretical and largely related to the modulation of cytochrome P450 enzymes by multi-botanical drug formulations, such as *Shengjiang Xiexin Decoction* (SXD) and *Banxia Xiexin* Decoction, as well as by polyphenol-rich dietary interventions (Manach et al., 2005; Izzo et al., 2009). In contrast, fiber-based therapies (e.g., partially hydrolyzed guar gum) and prebiotic formulations showed minimal potential for clinically relevant pharmacokinetic interactions (EFSA, 2010; Slavin, 2013), although rare cases of infection have been reported in severely immunocompromised patients (FAO/WHO, 2022; Sanders et al., 2010; McFarland, 2015).

Safety domains varied across interventions and included formulation variability and lack of standardization among botanical products (World Health Organization, 1999), limited pharmacokinetic and toxicological characterization of coded or polyherbal formulations (World Health Organization, 1999; WHO, 2004), and predominantly mild gastrointestinal adverse effects associated with dietary fibers (EFSA, 2010; Slavin, 2013). Interventions evaluated in oncological settings were considered to have greater clinical relevance due to their frequent concomitant use with chemotherapy or radiotherapy, highlighting the importance of proactive safety and interaction assessment despite the limited availability of direct clinical interaction data (World Health Organization, 1999; Izzo et al., 2009).

## Discussion

Electrolyte and fluid transport is a key function of the gastrointestinal tract, mediated by the intestinal epithelium through bidirectional exchange with luminal contents (Clemente-Suaréz et al., 2023; Negussie, 2022). Dysregulation of absorptive and secretory processes leads to diarrhea, characterized by excessive water loss in stool (Keely and Barrett, 2022). The pathophysiology of diarrhea is multifactorial and can include infections caused by viruses, bacteria, or parasites (Carmo et al., 2018), as well as alterations in the gut microbiota, immune hypersensitivity, genetic predispositions, allergens, bile acid malabsorption, side effects of medications (Keely and Barrett, 2022), and structural or functional intestinal disorders such as irritable bowel syndrome, inflammation, or malignancy (Gómez-Escudero and Remes-Troche, 2021).

Diarrhea is classified clinically as acute or chronic based on its duration. Acute diarrhea, typically caused by viral infections or poor diet, is usually self-limiting and of less clinical concern (Nemeth and Pfleghaar, 2022; Akhondi et al., 2025). In contrast, chronic diarrhea, defined as lasting longer than 4 weeks, poses a major diagnostic and therapeutic challenge and may severely impact a patient’s quality of life. Chronic diarrhea, which affects about 3%–5% of adults, is multifactorial (Gómez-Escudero and Remes-Troche, 2021) and may be divided into osmotic, secretory, inflammatory, dysmotility-related, or malabsorptive subtypes (Juckett and Trivedi, 2011). Treatment requires a comprehensive clinical examination, including detailed history and laboratory tests (Arasaradnam et al., 2018). These include quantification of fecal fat content, serum C-reactive protein, and fecal biomarkers such as calprotectin and lactoferrin to identify inflammatory or infectious causes (Camilleri; Sellin; Barrett, 2017; Arasaradnam et al., 2018).

Given the complexity and often limited efficacy of conventional pharmacological treatments, interest in natural products for treating acute and chronic diarrhea has increased due to their perceived safety and biological effects (Camilleri et al., 2016; Plaatjie et al., 2024). Giacosa et al. (2022) showed that *B. serrata* significantly reduced the duration of acute diarrhea compared with placebo. Previous studies indicate that *B. serrata* has anti-inflammatory and immunomodulatory effects, mainly by inhibiting 5-lipoxygenase activity and downregulating proinflammatory cytokines such as TNF-α and other interleukins (Giacosa et al., 2022). Although these mechanisms were not directly assessed in the study included in this systematic review, they provide a plausible biological rationale for the observed antidiarrheal effects. In addition, Entoban, a polytherapeutic formulation, has shown promising results in treating chronic diarrhea, comparable to or better than standard treatments such as metronidazole (Shakeel et al., 2016). These mechanisms may contribute to the observed antidiarrheal effects by reducing intestinal inflammation and preserving mucosal integrity.

As mentioned above, the treatment of chronic diarrhea is most effective when based on a thorough history and targeted diagnostic tests. Inflammatory diarrhea results from injury to the colonic mucosa, often due to inflammatory bowel disease (IBD) or infections such as *Clostridioides difficile* (*C. difficile*) (Gómez-Escudero and Remes-Troche, 2021). In a clinical study by Ried et al. (2020), administration of NC Gut Relief Formula led to improvement in diarrhea symptoms, which correlated with an increased abundance of beneficial gut microbiota, particularly *Clostridia* and *Lactobacillus* species. This study suggests that modulation of the gut microbiota may represent a promising alternative strategy for treating infectious diarrhea.

Botanical preparations have also demonstrated efficacy against parasitic infections. For example, *H. pubescens*, the main ingredient in the botanical product Amoebex, was effective in eradicating *E. histolytica* and relieving associated gastrointestinal symptoms, including diarrhea (Shah et al., 2016). Additionally, Berberine, a plant alkaloid, has shown broad-spectrum antimicrobial activity against pathogens such as *Escherichia coli*, *C. difficile*, *E. histolytica*, and *Vibrio cholerae*, and is also reported to have impact on clinical symptoms in patients with IBD (Di et al., 2020). The influence on outcomes of berberine has been attributed to the downregulation of NF-κB signaling in experimental models of IBS, leading to reduced mucosal inflammation and improved regulation of visceral sensitivity and intestinal motility (Yu et al., 2019). Additionally, berberine metabolites, including palmatine and jatrorrhizine, have shown *in vitro* antibacterial activity against several pathogenic species, such as *Bacillus cereus*, *Bacillus megaterium*, *Bacillus subtilis*, *Staphylococcus aureus*, *Staphylococcus epidermidis*, *Micrococcus lysodeikticus*, *Proteus vulgaris*, *Salmonella typhi*, and *E. coli* (Wang et al., 2017). These antimicrobial properties are consistent with the findings reported by Di et al., 2020.

In addition to what has already been described, diarrhea can also be a problem associated with antineoplastic treatment (Thomsen and Vitetta, 2018). Anticancer approaches, such as chemotherapy and radiotherapy alter the composition of the intestinal microbiota (Wang et al., 2016; Bukowski et al., 2020; Niculescu and Grumezescu, 2022) and cause damage to intestinal cells, leading to inflammation (Wang et al., 2016; Thomsen and Vitetta, 2018), changes in motility and ultimately diarrhea (Wang et al., 2016). Diarrhea associated with chemotherapy or radiotherapy is one of the most common side effects, along with nausea, abdominal cramps and changes in bowel movements. Patients also exhibit ulceration, reduced intestinal villi and crypts, and consequently a reduced capacity to absorb nutrients and electrolytes, factors that contribute to the occurrence of diarrhea (Huang et al., 2022).

Several studies included in this systematic review support these findings. Most describe the adverse gastrointestinal effects of chemotherapy, particularly the occurrence of diarrhea after treatment. For example, Lu et al. (2018) reported that 22.2% of patients with small cell lung cancer treated with irinotecan experienced grade 1 or two diarrhea, classified as mild or moderate according to the NCI-CTC (Common Terminology Criteria for Adverse Events). Delayed-onset diarrhea occurred in 15 patients (13.2%) receiving combination therapy with irinotecan, 5-fluorouracil, and L-leucovorin, with two patients (1.7%) developing severe late-onset diarrhea (Deng et al., 2017). In patients undergoing pelvic radiotherapy, severe diarrhea was observed from the start of treatment until day 28. The risk was even higher in patients who received combined radiotherapy and chemotherapy, such as 5-fluorouracil for colorectal cancer or cisplatin for gynecological tumors (Rosli et al., 2021).

Given the inter-individual variability in adverse effects of chemotherapy, such as irinotecan-induced diarrhea, recent studies have emphasized the importance of considering genetic polymorphisms in genes such as *UGT1A1*, *ABCB1*, and *SLCO1B1* that directly affect drug metabolism and pharmacokinetics, as shown in the studies by Deng et al. (2017) and Lu et al. (2018) included in this systematic review. Genetic polymorphisms, particularly in the *UGT1A*1 gene, play a significant role in the metabolism of irinotecan and the risk of associated toxicities. Polymorphisms such as UGT1A1*6 and UGT1A1*28 reduce the activity of the enzyme uridine diphosphate glucuronosyltransferase 1A1 (UGT1A1) to inactivate SN-38, the active metabolite of irinotecan, resulting in higher systemic SN-38 levels and an increased risk of severe diarrhea and neutropenia (Shimoyama, 2010; Atasilp et al., 2022). UGT1A1*6 is more commonly observed in East Asian populations, while UGT1A1*28 is more common in individuals of European and African descent (Su et al., 2023). These ethnic differences in allele distribution underscore the importance of population-specific genotyping strategies to better predict and manage toxicities associated with cancer treatments (Lauschke et al., 2019). As genotyping technologies advance, the potential for developing safer and more effective therapeutic strategies tailored to individual genetic and clinical profiles is increasing (Ho et al., 2020).

In addition, the natural products included in this systematic review, such as *Aloe vera* (Sahebnasagh et al., 2017; 2020) and *C. tetragonoloba* (Rosli et al., 2021), have shown promising results in reducing chemotherapy-related side effects, particularly the frequency of diarrhea, fecal urgency, and rectal bleeding. These effects may be attributed to PHGG, which has been reported to reduce diarrhea by enhancing mineral absorption and lowering blood cholesterol levels. Additionally, PHGG stimulates the production of short-chain fatty acids (SCFAs), particularly butyrate, promoting the growth of butyrate-producing bacteria and modulating the colonic microbiota, which is associated with beneficial health effects (Ohashi et al., 2015). PHGG may also help maintain intestinal barrier integrity by increasing SCFA production and modulating the expression of barrier-related genes, such as CLDN2 (Kono et al., 2023). Another botanical-derived antidiarrheal agent that has attracted considerable interest is crofelemer, an FDA-approved drug for the symptomatic relief of noninfectious diarrhea in adults with HIV/AIDS who are receiving antiretroviral therapy. Crofelemer is derived from the latex of *Croton lechleri* Müll. Arg [Euphorbiaceae] and exerts its antisecretory effects by modulating chloride ion transport through the cystic fibrosis transmembrane conductance regulator (CFTR) and calcium-activated chloride channels (CaCC). More recently, clinical studies have reported promising outcomes with crofelemer in patients experiencing chemotherapy-induced diarrhea, suggesting its potential as a supportive therapeutic option in oncology (Greene et al., 2021; Jacob et al., 2023). The study by Rosli et al. (2021) reported a reduction in the incidence of diarrhea; however, no specific mechanism was identified for this effect. Moreover, no significant differences were observed in quality of life, as assessed with the EORTC QLQ-C30 questionnaire. Notably, similar compounds have also been investigated in non-oncologic gastrointestinal disorders, suggesting broader therapeutic relevance. Interventions such as NC Gut Relief Formula (Ried et al., 2020) and Amoebex (Shah et al., 2016) have shown benefits in treating constipation, abdominal discomfort, and diarrhea.

Taken together, these results highlight the potential of natural products as complementary strategies for maintaining gastrointestinal health in diverse clinical contexts. These findings underscore the value of integrating natural adjuvant therapies into modern clinical practice, particularly when combined with genetic screening tools that enable more precise, patient-specific treatment approaches.

### GRADE certainty of evidence and statistical significance

Although several included studies reported statistically significant improvements in diarrhea-related outcomes in both oncological and non-oncological settings, these findings should be interpreted with caution. Statistical significance alone does not imply high certainty of evidence or guarantee clinically meaningful effects. Using the GRADE framework, certainty of evidence was assessed at the outcome level and was predominantly rated as low or very low. These ratings primarily reflected methodological limitations across the included studies, including risk of bias, imprecision due to small sample sizes, inconsistency or indirectness of the evidence, and potential publication bias. Consequently, confidence in the estimated treatment effects remains limited, and the true effect may differ substantially from the observed estimates.

In oncological settings, reductions in the frequency and severity of chemotherapy- or radiotherapy-associated diarrhea were reported with relative consistency. However, important methodological limitations were common, including small sample sizes, heterogeneous outcome definitions, and insufficient reporting of blinding and allocation concealment. Several outcomes were also informed by single studies or a very limited number of trials, increasing uncertainty around effect estimates and susceptibility to publication bias.

Comparable limitations were observed in non-oncological populations. Despite statistically significant findings, variability in comparators, inconsistencies in diagnostic criteria, heterogeneity in formulations and dosing regimens, and incomplete reporting of statistical methods hindered direct comparisons and reduced confidence in the observed effects.

Across both contexts, imprecision was frequent due to small-scale, single-center designs and the absence of confidence intervals in several studies. Indirectness also affected multiple outcomes, as some investigations focused on highly specific populations or clinical scenarios, limiting generalizability. Publication bias was considered plausible given the limited number of available studies and the predominance of favorable findings in research areas susceptible to selective reporting.

Overall, although the available evidence suggests a favorable association between the evaluated interventions and improvements in diarrhea-related outcomes, certainty was predominantly low according to GRADE criteria. This reflects not only the methodological quality of individual studies but also the scarcity of robust, reproducible evidence. Accordingly, these findings should be viewed as indicative rather than definitive, underscoring the need for well-designed randomized controlled trials with larger sample sizes, standardized outcome measures, and transparent reporting to support more reliable clinical conclusions.

### Implications and mechanistic hypotheses

The subgroup analysis offers important insights into potential mechanisms underlying the clinical effects of natural products on diarrhea. In oncological settings, reductions in chemotherapy- or radiotherapy-induced diarrhea are primarily associated with interventions that modulate the gut microbiota, reduce inflammation, and support epithelial barrier integrity, reinforcing the biological plausibility of these strategies. In contrast, in non-oncological contexts, symptom improvement may be more directly related to the antimicrobial and anti-inflammatory properties of specific botanical drugs.

Despite these emerging patterns, the consistently low GRADE certainty across both subgroups emphasizes that statistically significant findings should not be interpreted as evidence of high confidence in efficacy or safety. Nevertheless, this exploratory synthesis generates clinically relevant, testable hypotheses. Future trials may benefit from stratification by diarrhea etiology and intervention class to better delineate mechanism-specific effects, promote outcome standardization, and incorporate structured safety evaluations, including assessment of potential herb–drug interactions, particularly in oncology populations.

Collectively, these findings show that even in the absence of meta-analytic pooling, structured subgroup analyses can reveal mechanistic signals and inform hypothesis-driven research, while maintaining transparency regarding methodological limitations and the certainty of the evidence.

### Interpretation of safety and interaction findings

This review goes beyond clinical efficacy by incorporating safety considerations, mechanistic plausibility, and potential herb-drug interactions, drawing on external pharmacological and toxicological evidence. Although several interventions showed favorable effects on diarrhea-related outcomes, the available safety and interaction data remain largely indirect, especially for multi-component botanical formulations.

In oncology settings, the potential for herb-drug interactions requires particular attention, as many botanical drugs may theoretically modulate cytochrome P450 enzymes involved in the metabolism of chemotherapeutic agents. While such interactions were not directly observed in the included trials, their biological plausibility highlights the need for cautious interpretation and ongoing clinical vigilance. In contrast, microbiota-targeted interventions, such as prebiotics and fiber-based supplements, demonstrated comparatively favorable safety profiles and minimal interaction potential, supporting their use as adjunctive strategies when administered under appropriate clinical supervision.

The limited pharmacokinetic and toxicological characterization of several botanical drugs likely contributed to the predominantly low certainty of evidence identified through the GRADE framework. Notably, statistically significant clinical findings should not be interpreted as evidence of robust safety or interaction profiles. Future research should prioritize pharmacokinetic evaluation, formulation standardization, and structured safety reporting, particularly in oncology populations, where polypharmacy and narrow therapeutic indices further increase the clinical relevance of interaction risk.

### Implications for clinical practice, with emphasis on oncology

In oncology, diarrhea, particularly when induced by chemotherapy or radiotherapy, is a clinically significant complication that can lead to treatment interruption, dose modification, and reduced quality of life. Available evidence suggests that certain botanical drug formulations and functional supplements may provide adjunctive benefits in reducing the severity and frequency of diarrhea through biologically plausible mechanisms, including anti-inflammatory activity, modulation of intestinal motility, and support of epithelial barrier integrity. However, the certainty of evidence supporting these interventions remains consistently low. Therefore, none of the reviewed products can be recommended as standard therapy in oncology care. Their use should be limited to supportive roles, preferably within structured integrative oncology frameworks and under close medical supervision. This caution is especially important given the potential for herb-drug interactions identified through pharmacokinetic and toxicological evidence, especially in patients receiving anticancer agents with narrow therapeutic indices or metabolism dependent on cytochrome P450 enzymes.

In non-oncological populations, including those with infectious, functional, and chronic diarrhea, the evidence appeared somewhat more consistent regarding symptom improvement. Nevertheless, substantial heterogeneity in study design, formulations, dosing strategies, and outcome definitions resulted in similarly limited certainty. Although several interventions showed clinically meaningful effects, the lack of standardized efficacy endpoints, limited long-term safety data, and insufficient characterization of interaction risk preclude firm clinical recommendations.

Overall, these findings highlight the need for cautious, individualized clinical decision-making and underscore the importance of future trials that incorporate rigorous safety evaluation, formulation standardization, and explicit assessment of pharmacokinetic and herb-drug interactions, priorities that are especially critical in clinically vulnerable populations such as oncology patients.

### Strengths and limitations

This review suggests a potential beneficial role for natural products in managing diarrheal conditions; however, several methodological limitations across the included studies weaken the overall strength and reliability of the evidence. Only a subset of studies used randomized, double-blind designs, while others were single-arm or open-label, increasing susceptibility to bias, placebo effects, and subjective outcome assessment. Small sample sizes, heterogeneous treatment durations, and the use of non-standardized instruments to evaluate diarrheal symptoms further limited comparability and reduced confidence in the findings.

Additional limitations include the absence of long-term follow-up and insufficient reporting of safety and tolerability outcomes. Many studies provided minimal information on adverse events, dose-related effects, and potential interactions with concomitant medications–an issue of particular relevance when natural products are used alongside conventional pharmacotherapies. Furthermore, aspects related to product quality, standardization of active constituents, and regulatory oversight were rarely addressed, restricting a comprehensive assessment of risk–benefit profiles.

A key strength of this review is the transparent application of the GRADE framework despite the absence of meta-analysis, demonstrating that structured certainty assessment remains feasible even in the context of heterogeneous evidence. The inclusion of both oncological and non-oncological settings also enhances clinical interpretability while preserving outcome-level rigor. Nevertheless, reliance on published data, the plausibility of publication bias, variability in intervention formulations, and the predominance of small-scale, single-center studies limited the feasibility of quantitative synthesis and contributed to the overall low or very low certainty of evidence.

Taken together, these limitations underscore the need for well-designed, adequately powered randomized controlled trials incorporating standardized outcome measures, comprehensive safety reporting, and clear documentation of product quality and regulatory compliance. Such efforts are essential to better define the clinical role of natural products in diarrhea management.

## Conclusion

This review highlights that diarrhea may result from infectious and inflammatory conditions, as well as from cancer therapies such as chemotherapy and radiotherapy, which are often associated with intestinal barrier disruption and microbiota imbalance. The natural agents evaluated in the included studies showed potential benefits, including reduced diarrhea duration, improved stool consistency, and enhanced overall gastrointestinal function. However, when assessed using the GRADE framework, the certainty of evidence was mostly low or very low, reflecting methodological limitations, imprecision, heterogeneity, and potential publication bias. Therefore, although current evidence suggests a favorable effect, confidence in these estimated benefits remains limited. Future well-designed randomized controlled trials with adequate sample sizes, standardized outcome measures, and transparent reporting are needed to clarify underlying mechanisms and strengthen the certainty of the evidence supporting the use of natural agents as complementary strategies in diarrhea management.

## Data Availability

The original contributions presented in the study are included in the article/Supplementary Material, further inquiries can be directed to the corresponding author.
